# Community-Based Family Workshop Intervention Improved the Social Adaptation of Left-Behind Children in Rural China

**DOI:** 10.3389/fpubh.2020.506191

**Published:** 2020-12-11

**Authors:** Xiu Zhang, Mengjie Li, Li Guo, Yanna Zhu

**Affiliations:** ^1^Department of Maternal and Child Health, School of Public Health, Sun Yat-sen University, Guangzhou, China; ^2^Department of Maternal and Child Health and Sun Yat-sen Global Health Institute, School of Public Health and Institute of State Governance, Sun Yat-sen University, Guangzhou, China

**Keywords:** family workshop, community-based intervention, left-behind children, social adaptation, rural China

## Abstract

**Purpose:** With the rapidly developing economy in China, there are more than 50 million left-behind children (LBC) in rural China, whose social adaptation has become a public concern. Thus, the current study aimed to investigate the effect of community-based family workshop intervention on social adaptation among rural LBC in China.

**Methods:** A cluster randomized trial was conducted with 104 LBC, in which 66 LBC-caregivers dyads received guidance from community-based family workshop for 6 months, while the controls (LBC *n* = 38) received routinely parenting guidance. Social adaptation of the children was assessed by the Strength and Difficulties Questionnaire (SDQ) at the beginning and the end of the intervention.

**Findings:** Compared with controls, results showed remarkable improvement on emotional symptoms (*P* = 0.050), peer problems (*P* = 0.050), and total difficulties score (TDS, *P* = 0.040) in the intervention group, especially those aged 3–6 years. Moreover, SDQ score of TDS (*P* = 0.039), peer problems (*P* = 0.013), and hyperactivity–inattention (*P* = 0.023) decreased after intervention in boys aged 3–6 years, while emotional symptoms (*P* = 0.048) in girls. Finally, improvement on peer problems (*P* = 0.005) was observed in participants with high TDS.

**Conclusions:** The findings suggest that community-based family workshop intervention can improve social adaptation of rural LBC. Moreover, this effect was different in boys and girls and also affected by age and baseline total difficulties. The results indicated that community-based family workshop intervention can be implemented in rural China to improve mental and behavioral health among LBC in the future.

## Introduction

China has become one of the largest economies since the reform and opening-up policy started in 1978 (1). Along with the unceasingly thorough development of this policy and the accelerating process of urbanization in China, millions of rural surplus labors have transferred to urban areas for employments and opportunities in the last three decades. However, under the strict restriction of the urban–rural dual structure, most of the migrants cannot get urban household registration, which means they do not have access to the same social services as urban residents do, including healthcare, education, and social security. Thus, most of them chose to leave their minor children at rural hometown with single parent, grandparents, or other relatives (2). In China, *left-behind children* (LBC) was defined as children and adolescents younger than 17 years and who have lived with a single parent or extended family at hometown for more than half a year while one or both of their biological parents are away for work (3). However, with the rapid development of the economy in China, the population migration rate is increasing gradually, especially after 2000. More and more rural laborers migrate to the big cities for better job opportunities, resulting in the soaring number of LBC in China (4). In 2015, there are more than 68.7 million LBC in China, accounting for 25.39% of total children. Above all, 79.9% of the LBC live in rural areas, and nearly three-quarters of them are younger than 11 years (5). The proportion of LBC younger than 5 years has increased to 40.34% (2015) from 27.05% (2005) in the past decade (5, 6). As is known to all that, it is the parents who teach the children what is appropriate for them and show them how to be a member of this society for the first time (2, 7). Unfortunately, to LBC, parental absence may result in a lack of parental guidance, face-to-face communication, emotion care or support, supervision, and low frequency of interactions (8), which is associated with poorer child well-being, including mental health problems (9, 10), behavioral problems (2, 11, 12), and interpersonal relationship problems (13), which could increase the difficulty for them to adapt to the society (14, 15).

Social adaptation occurs in the process of socialization. For children, it refers to the ability of the children to adjust their behavioral habits or attitudes to be more age-appropriate so as to adapt to the living environment (15). The adaptation includes the cultivation of healthy lifestyles and self-management ability, the prosocial behavior habit that conforms to the social norm, the ability of self-consciousness appropriate to the current social roles, and social communication ability (16, 17). Previous studies indicated that compared with non-LBC, school-aged LBC are more likely to have more sensitive, hostile, and paranoid interpersonal relationship (13), as well as higher rates of negative emotional experience such as depression (9), anxiety (10), and loneliness (9). Prior studies showed that LBC who had an experience of separation at a younger age or for a longer time were more likely to suffer from behavioral or psychological problems (2, 11). For instance, those who were separated from both parents were faced with more difficulties during social adaptation compared with those with a single parent absent (18). Besides, studies reported that girls tended to have more emotional problems, whereas boys had more behavioral symptoms when left behind (12). A large amount of resources will be needed in the future, if the situation continues, which will become a burden to the society. Therefore, more efforts are needed to improve LBC's social adaptation and to promote their health development both mentally and physically.

Processes of social adaptation are inevitably interwoven with the style of child-rearing, including parent–child interaction, parent–child relationship, and parent behaviors and attitudes (15). However, the parent–child interaction of LBC is often characterized by low level of parental involvement, poor supervision of offspring (8), and worse quality of parent–child relationship and communication (19). However, despite the high levels of public concern about the development of LBC in China, few studies have investigated the social adaptation of LBC. Besides, most of previous intervention studies were conducted in school-aged children or in adolescents and place emphasis on emotional symptoms or behavioral problems (20), while few intervention researches aimed at solving different problems according to the different characteristics of development in different ages in LBC, especially those younger than 10 years. Luckily, clear evidences showed that group-based parenting intervention for preschool children that was underpinned by social learning theory (21) can improve child development of emotion regulation ability, behavioral adjustment (22–24), and social skills (25). Niec et al. (26) found that parent–child interaction therapy, which was based on social learning theory, attachment theory, and traditional play therapy, could reduce the destructive behaviors and increase the prosocial behavior of preschoolers. Bywater et al. (27) also found that group-based parenting program held in communities that was underpinned by cognitive and behavioral theory could reduce the incidence of behavioral problems of children with conduct disorder and enhance their emotional regulation ability. Besides, according to the triadic reciprocal determinism illustrated by Bandura, behaviors are affected by person/cognition and environment (21). Most human behaviors are learned by observing and imitating others, and the environment plays a decisive role in this process (21). Meanwhile, evidences showed that supportive community networks can improve children's social skills such as assertiveness, communication, social interaction, and cooperation (28). Along with the unceasingly thorough development of China new rural reconstruction, the environment in rural communities is gradually improving. It is urgent to explore an effective community intervention for children in accordance with the rural community environment in China. However, few researches focused on community-based intervention in LBC in China.

Thus, a community-based family workshop intervention underpinned by cognitive behavioral theory (26, 29) was adopted in the current study, we aimed to assess the effectiveness of the intervention on improving the social adaptation among LBC aged 3 to 9 years in rural Guangdong province, where there is the largest number of rural LBC in South China (5). We hypothesized that this workshop intervention in rural LBC may improve their social adaptation of behaviors, emotions, and relationship. Considering the fact that children usually start primary school at the age of 6 years in China, when the relationship with peers and elders becomes a more important part to his social relationship. Based on the theory that psychological cognition development in children has close relationship with age and sex (30), we also postulated that the intervention effect on child development may have age and sex differences.

## Materials and Methods

### Participants and Study Design

The intervention program was conducted from August 2014 to March 2015 in 15 rural communities in Guangdong Province, South China. Participants including caregiver–child dyads were enrolled from 15 communities in Guangdong Province, in August 2014. Recruiting posters were distributed, and participants voluntarily called the local staff for registration. Inclusion criteria included (1) children aged 3–9 years and one of their main caregivers, (2) rural household registration, (3) children were left behind by one or two migrant parents for at least 6 months, and (4) signed informed consent by caregiver voluntarily. Exclusion criteria contained (1) both child and parents/caregivers who had a history of serious mental or neurological systemic disease or with obvious physical disability. Considering that most of these group leaders were semiprofessional, their expertise was not enough to work with these kinds of children or adults with serious mental or physical disorders in the group. Besides, the program contained a lot of sports and communication; we had to make sure every child can participate in the activity smoothly. For these reasons, we made these exclusion criteria. Finally, a total of 104 LBC were recruited into the study, allocated to intervention group (*n* = 66, mean age 6.9 years) and control group (*n* = 38, mean age = 7.1 years) (Table 3). Written informed consents were obtained from statutory guardians of the children before the trial started.

The study was designed as a community-based effectiveness trial with a duration of 6 months. During the study process, children social adaptation including behaviors, emotions, and relationship were assessed by the Strength and Difficulties Questionnaire (SDQ) (31) at baseline and after 6 month intervention. The protocol of this study was designed by child experts (four women and two men, the average age was 42 years) in psychology, behavioral science, and child development. This study was conducted in accordance with the Declaration of Helsinki, and the protocol was approved by the institutional research ethics committee of Sun Yat-sen University, as well as the institutional review board of Guangdong Women's Federation, an administration of the government.

### Assignment Method

A two-step process was used to identify communities. First, we stratified these communities into five layers based on geographic location (east, south, middle, west, and north). Three rural communities with a relatively large number of LBC were chosen in the same prefecture-level city in each location. Second, a simple randomization method and random number table were used to allocate these three communities at a 2:1 ratio to an intervention and a control group, because we hope as many LBC as possible could receive the intervention. Finally, we got 10 intervention communities and 5 control communities. Random assignment was done by one researcher and an official in Women's Federation who were not involved in the direct implementation of the program.

**Figure 1:**
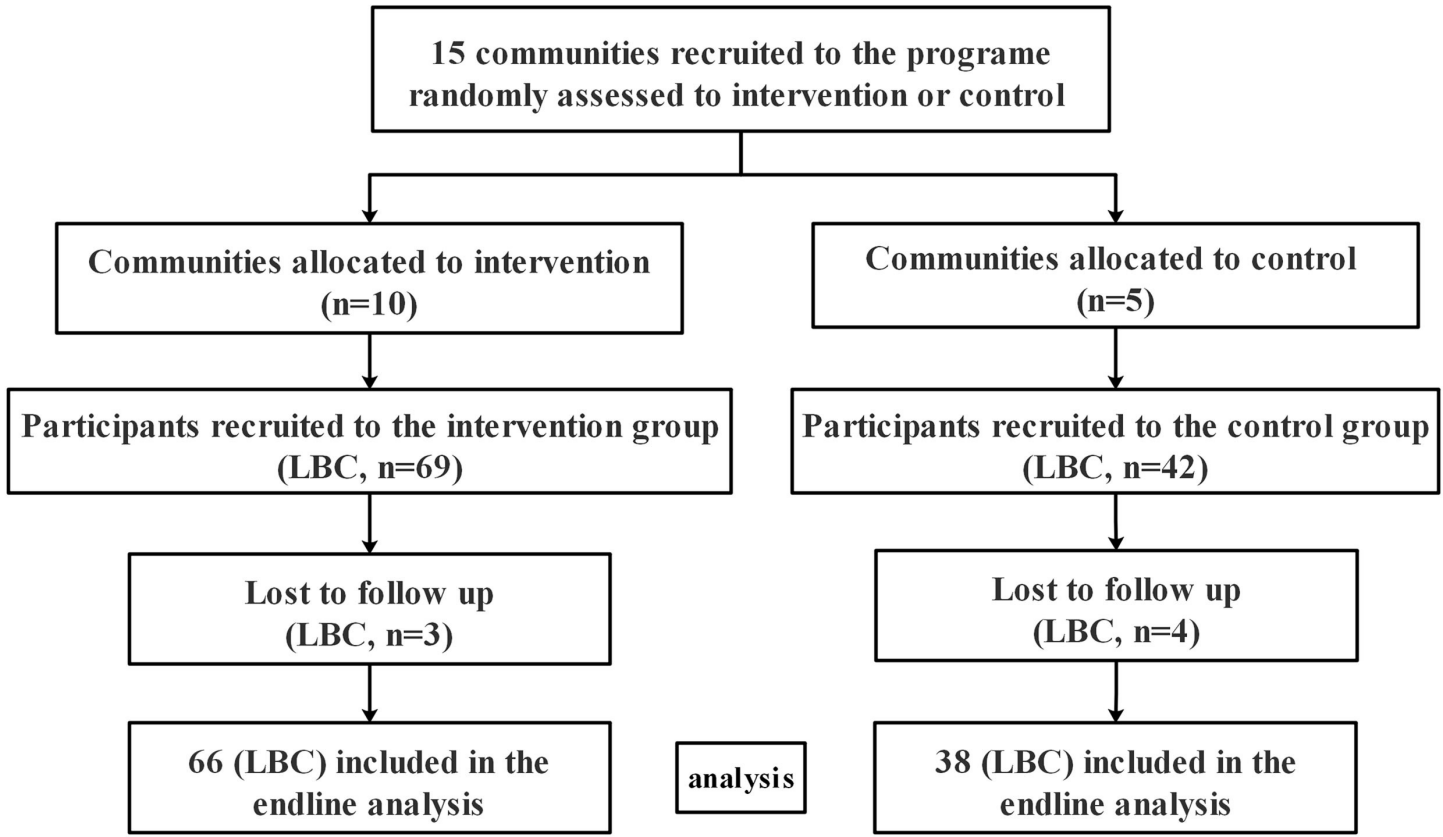
Participant flow.

Based on an α error of 0.05 and a power of 90% (two-sided test), the power analysis suggested that a sample of 64 participants would reach an effect size (ES) of 0.5. Considering the attrition of 20%, the sample size rose to 80. Considering that each workshop contained 6 to 8 children, 111 LBC were recruited to the program at baseline, and 104 of them finished the program. The participant flow is shown in Figure 1.

### Setting and Personal

This study was performed in Kids Activity Center in rural communities, the Child-Friendly Community, specializing in offering intervention, activities space, and services to the children. All of the Kids Activity Center in the intervention communities had four rooms (reading room, handcraft room, computer room, and multifunctional activity room), a playground (with wings, seesaws, climbing rack, basketball court, etc.), and teaching videos. The facilities included books, computers, video players, and sports equipment, which were provided by the Heungkong Charitable Foundation unity. Besides, the intervention was delivered by 10 well-trained group leaders, recruited by Guangdong Women's Federation. They were recruited according to these criteria: (1) had the education background in teaching, pedagogy, or psychology; (2) had attended professional training courses of psychological counseling or social service work; and (3) had the experience of leading group activities or group consulting. Meanwhile, each Kids Activity Center had a manager (local women's director) responsible for daily management.

### Intervention Procedures

In the trial, both the intervention group and the control group were invited to take part in the regular activities organized by each community, such as manual activities, book reading, physical exercise, watching the teaching video, etc., or popular science propaganda or parenting education (such as parenting and nutrition propaganda) during September 2014 to February 2015.

Meanwhile, the intervention group received guidance from community-based family workshop for 6 months (from September 2014 to February 2015). Both LBC and one of their main caregivers were required to take part in the community-based family workshop together every time. The form of the workshop was group-based integrated intervention with 4 stages and 12 sessions. Each session had 2 h, which was conducted every 2 weeks. Besides, a group consulting form (32) was used in the intervention to improve relationships between caregivers and children and the social–emotional competencies of children. A series of structured activities based on play theory (26) were used in the sessions. Considering that the development of children's social–emotional competencies is a developmental process in which children obtained different skills at different ages (30), the group was divided by ages (3–6 and 7–9 years). Caregiver is defined as the one who provides the child most of the care and social interaction (33). In the present study, main caregivers consist of grandparents (54.5%), mother (34.8%), father (4.5%), brother (4.5%), and aunt (1.5%) (Table 3).

The integrated intervention included 4 stages and 12 sessions: preparation (two sessions), exploration (four sessions), practice (four sessions), and consolidation (two sessions). The periodic goals and structural activities at each stage of LBC aged 3–6 and 7–9 years are shown in Tables 1, 2. The preparation stage aimed at establishing a sense of trust, increasing team cohesion through ice-breaking activities, and constructing the group (including group name, slogan, rules, etc.) and then moved to the exploration stage, which focused on exploring the patterns of parent–child interactions, communications, attachment, and children's behavior, by a series of interactive activities based on cognitive–behavioral theory (21) (3–6 years: role play, what kind of person I am in the eye of my parents or my child, parent–child interactions, hand in hand; 7–9 years: role play, my self-portrait, caregivers and child swap roles, etc.). The practice stage of 3–6 years focused on effective communication skills learning (love, respect, empathic, patient, non-judgmental) and secure attachment behaviors practicing (sensitive to children's need and response correctly to them, give them emotional support timely). Targeted activities (e.g., role play, story-telling, parent–child handcrafting, drawing, or concert) were taken to help caregivers practice and generalize these parenting skills. As for LBC aged 7–9 years, the periodic goal was effective communication skills learning, rational expression of emotion practicing, and learning to cooperate through structured activities such as role play, painting blindfolded, three-legged race, or sports meeting. Finally, the consolidation stage aimed at helping the group members make a review and summary, so as to maintain and enhance the skills as well as saying goodbye to other group members.

**Table 1:** Periodic goals and structural activities at each stage in the community-based family workshop for LBC aged 3–6 years and their caregivers.

**Stage**	**Periodic goals**	**Structured activities**	**Sessions**
One: Preparation	1. Establishing a sense of trust 2. Increasing team cohesion	Self-introduction Ice-breaking activities: throwing the handkerchief Chicken vs. eagle	Two sessions
Two: Exploration	1. Exploring the patterns of parent–child interactions, communications, and attachment 2. Exploring the patterns of preschooler's behavior	Role play What kind of person I am in the eye of my parents or my child Parent–child interactions Hand in hand	Four sessions
Three: Practice	1. Effective communication skills learning 2. Practicing secure attachment behaviors	Tell a story Role play Parent–child handcrafting, drawing or concert	Four sessions
Four: Consolidation	1. Make a review and summary 2. Maintain and enhance the skills	Big feet and small feet Parting gifts	Two sessions

**Table 2:** Periodic goals and structural activities at each stage in the community-based family workshop for LBC aged 7–9 years and their caregivers.

**Stage**	**Periodic goals**	**Structured Activities**	**Sessions**
One: Preparation	1. Establishing a sense of trust among team members 2. Increasing team cohesion	Self-introduction Who is younger? Throwing the handkerchief Chicken vs. eagle	Two sessions
Two: Exploration	1. Exploring the patterns of parent–child interactions, communications 2. Exploring the patterns of school-aged children's behavior	Role play Myself-portrait Caregivers and child swap roles	Four sessions
Three: Practice	1. Effective communication skills learning 2. Rational expression of emotion practicing 3. Learning to cooperate	Role play Painting blindfolded Three-legged race Sports meeting	Four sessions
Four: Consolidation	1. Make a review and summary 2. Maintain and enhance the skills	The unique me The caregiver and child flatter each other Farewell party	Two sessions

After the activities, a discussion on experience and information sharing would be held by the group leader in half an hour aiming to help the caregivers better understand their weakness and reinforce positive behaviors. All of these caregivers in the workshop were encouraged to share their feelings, experiences, information, and confusions they had during the game. At the end of the session, the group leader would point out the ineffective parenting behaviors (e.g., critical, judgment, impatient, rude, negative physical contact). After the sessions, the caregivers were encouraged to share experiences with other family members and use the skills not only at community settings but also at home.

In the whole intervention process, the most important work of the group leader was to create a free, equal, safe, and inclusive atmosphere, so that each member can feel respect and support and express themselves freely. Meanwhile, the leader was a group member at the same time and interacted with other group members.

### Measures

#### Child Social Adaptation

The SDQ (31), including 25 items, was originally developed by Goodman. It aims to assess behaviors, emotions, and relationship in children aged 3 to 17 years (34, 35). The questionnaire is divided into five subscales: emotional symptoms, conduct problems, hyperactivity–inattention, peer problems, and prosocial behaviors, with five items in each subscale, ranging from 0 to 10. The first four subscales' score constitutes a total difficulties score (TDS), ranging from 0 to 40, and a TDS > 14 was considered to be at a high score range (35). The SDQ has been proven to have good reliability and validity in Chinese culture (35–37). The Cronbach α for the whole Chinese version of SDQ was 0.84 (37). Thus, in the current study, the parent report Chinese version of SDQ (35) was adopted and was filled out by the main caregiver.

### Quality Control of the Intervention

To enhance the homogeneity of the intervention, 1 week before the intervention started, all of these group leaders and managers were trained by the Guangdong Women's Federation staff and two authors (LMJ and GL) for 20 h. The training was based on The Instruction Manual of Working with Children in Rural Communities, which was the guideline of the intervention. The training included workshops, intervention process, and intervention methods for children of different ages. Considering that most of these group leaders were semiprofessional, regular supervision was provided to them by a psychological counselor with Chinese national qualifications. Meanwhile, the group leader should also contact their supervisor if anything unexpected happens, such as the loss of a family in the group or the unexpected emotion breakdown of some group members, etc. Group leaders adhered to the protocols of content and teaching methods. All of these group leaders and managers were paid by the project monthly.

In order to monitor the quality of the intervention, each of the papery archives including activities records (time, place, contents, frequency, participation, etc.), child records (basic demographic information), and work diary was established in all of these 10 sites before the intervention started. Besides, an assessment team consisted of the researchers who had no direct role in the implementation of the intervention, supervised the progress by monthly on-site inspection, and evaluated the community monthly summary (attendance rate of the sessions, problems in previous sessions, feedback information, etc.) submitted by the group leaders. The supervise scope included viewing textual archives, checking activity forms and involvement. Then the supervisors gave the feedback in a structured monitoring form.

### Data Collection and Statistical Analysis

Local Women's Federation staffs with years of experiences of field questionnaire survey were recruited. Before the study started, 1 week accentuation training for data collection was given uniformly by Guangdong Women's Federation and the project team to them. All of these staffs were not involved in the direct implementation of the program. The baseline data were collected over the course of 2 weeks in August 2014 and end-line data in March 2015. All these data were collected and rechecked in the kids-activity center to maintain the data quality.

The data were entered by Epidata 3.1 and analyzed by the Statistical Package for the Social Science (SPSS) (version 21.0; SPSS Inc., Chicago, IL, USA). All of the families recruited to the program were included in the baseline analysis. χ^2^ test and the unpaired Student *t*-test were adopted to describe the baseline characteristics. In addition, the intervention effect was assessed by repeated measures analysis of variance (ANOVA) with the adjustment of community clustering effects. Then the participants were divided into two groups by age (3–6 and 7–9 years) so as to examine the intervention effects separately. Besides, we tested the effectiveness of the intervention to boys and girls in the program separately. Finally, we tested the intervention effects by stratifying the participants into low-TDS (range, 0–14 scores) and high-TDS (range, 15–40 scores) (35) groups based on the TDS at baseline. All these results were expressed as mean ± standard deviation, ES, and 95% confidence interval (CI). Significance was specified at *P* < 0.05, two-sided test.

## Results

### Baseline Characteristics

Basic characteristics of the participants are displayed in Table 3. Seven participants were lost because of caregiver's illness or their moving away from the present communities (three of the intervention group and four of the control group). Finally, 104 LBC (93.6%) completed the final assessment at the end of the intervention (66 of intervention group, 38 of control group). On average, the main caregiver in the intervention group attended 10.8 group sessions, with 80% of main caregiver attending 10 sessions or more. No side effect was observed during the intervention period. At baseline, no significant differences were found between the two groups in demographic characteristics, including sex, age, only child, separating age, family monthly income, educational level of both parents, and the SDQ subscales scores (all *P* > 0.05).

**Table 3:** Baseline Characteristics of Participants in the FW and Control Groups^a^.

**Variables**	**FW (*n* = 66)**	**Control (*n* = 38)**	**χ^2^ or *t***	***P***
**Child**				
**Sex**, ***n*** **(%)**				
Boy	36 (54.5)	17 (44.7)	0.928	0.335
Girl	30 (45.5)	21 (55.3)		
**Only child**, ***n*** **(%)**				
Yes	9 (13.6)	7 (18.4)	0.424	0.515
No	57 (86.4)	31 (81.6)		
Age, years, mean (SD)^b^	6.9 (2.1)	7.1 (1.7)	−0.631	0.530
**Separating age**, ***n*** **(%)**				
0–3 years old	36 (54.5)	20 (52.6)	0.597	0.742
3–6 years old	23 (34.8)	12 (31.6)		
6–10 years old	7 (10.6)	6 (15.8)		
**Main caregiver when left behind**, ***n*** **(%)**				
Grandparents	36 (54.5)	17 (44.7)	5.422	0.247
Father	3 (4.5)	5 (13.2)		
Mother	23 (34.8)	16 (42.1)		
Brother of sister	3 (4.5)	0 (0.0)		
Relatives	1 (1.5)	0 (0.0)		
**Family**				
**Monthly income (RMB, %)**				
<2,000	18.8	28.9	3.896	0.420
2,000–5,000	59.4	47.6		
5,000–8,000	14.1	7.9		
8,000–12,000	0.0	0.0		
>12,000	1.6	2.6		
Unknown	6.3	13.2		
**Father's educational level**, ***n*** **(%)**				
None/primary	2 (3.0)	3 (7.9)	1.181	0.277
Secondary (middle school/vocational school)	61 (92.4)	31 (81.6)		
University or above	3 (4.5)	4 (10.5)		
**Mother's educational level**, ***n*** **(%)**				
None/primary	5 (7.6)	2 (5.3)	0.213	0.899
Secondary (middle school/vocational school)	56 (84.8)	33 (86.8)		
University or above	5 (7.6)	3 (7.9)		
**SDQ subscales scores, mean (SD)^b^**				
Emotional symptoms	2.79 (1.80)	2.34 (1.89)	1.193	0.236
Conduct problems	2.05 (1.71)	1.87 (1.53)	0.527	0.599
Hyperactivity–inattention	4.14 (1.81)	3.89 (2.17)	0.601	0.543
Peer problems	3.27 (1.56)	2.87 (1.53)	1.285	0.202
Total difficulties score	12.24 (4.78)	10.97 (5.49)	1.235	0.220
Prosocial behavior	6.30 (2.05)	6.58 (2.31)	−0.632	0.529

*FW, family workshop; SDQ, Strength and Difficulties Questionnaire*.

a *Data were assessed by χ^2^ test for categorical variables*.

b *Assessed by the unpaired Student t-test*.

### Effects of Intervention on SDQ Scores in Total LBC

Changes of SDQ scores after 6 month intervention in total LBC are shown in Table 4. Compared with the control group, the subscale scores of SDQ including total difficulties score (TDS) [−2.971 (95% CI, −5.797 to −0.146), *P* = 0.040], emotional symptoms [−1.073 (95% CI, −2.147 to 0.002), *P* = 0.050], and peer problems [−0.774 (95% CI, −1.547 to −0.002), *P* = 0.050] significantly decreased in total participants of intervention group after the intervention. Although no statistical significance was observed, subscale scores of TDS [−4.248 (95% CI, −8.499 to 0.003), *P* = 0.05] and peer problems [−0.986 (95% CI, −1.999 to 0.028) *P* = 0.056] were prone to decrease in girls after intervention. No significant changes of SDQ scores were observed in boys after the intervention (all *P* > 0.05) (Table 4).

**Table 4:** Comparison of SDQ scores between FW and control groups after 6 month intervention^a^.

**Subscale scores**	**Total (*****n=*** **104)**				***P***	**ES [95% CI]**	**Boys (*****n=*** **53)**				***P***	**ES [95% CI]**	**Girls (*****n=*** **51)**				***P***	**ES [95% CI]**
	**FW (*****n=*** **66)**		**Control (*****n=*** **38)**				**FW (*****n=*** **36)**		**Control (*****n=*** **17)**				**FW (*****n=*** **30)**		**Control (*****n=*** **21)**			
	**Baseline**	**6 months**	**Baseline**	**6 months**			**Baseline**	**6 months**	**Baseline**	**6 months**			**Baseline**	**6 months**	**Baseline**	**6 months**		
Emotional symptoms	2.79 (1.80)	2.14 (1.85)	2.34 (1.89)	2.76 (2.52)	0.050^*^	−1.07 [−2.15, 0.00]	2.89 (1.89)	2.14 (2.00)	2.41 (2.12)	2.53 (2.83)	0.333	−0.87 [−2.65, 0.92]	2.67 (1.71)	2.13 (1.67)	2.29 (1.74)	2.95 (2.29)	0.070	−1.20 [−2.50, 0.10]
Conduct problems	2.05 (1.71)	2.15 (1.98)	1.87 (1.53)	2.37 (2.30)	0.427	−0.39 [−1.37, 0.59]	1.94 (1.55)	2.31 (2.04)	2.12 (1.65)	2.18 (2.04)	0.636	0.30 [−0.97, 1.58]	2.17 (1.91)	1.97 (1.92)	1.67 (1.43)	2.52 (2.52)	0.172	−1.06 [−2.59, 0.47]
Hyperactivity-inattention	4.14 (1.81)	3.48 (2.02)	3.89 (2.17)	3.97 (2.26)	0.112	−0.73 [−1.64, 0.17]	4.14 (1.71)	3.58 (1.90)	4.18 (2.48)	4.06 (2.56)	0.467	−0.44 [−1.64, 0.76]	4.13 (1.94)	3.37 (2.17)	3.67 (1.91)	3.90 (2.05)	0.159	−1.01 [−2.42, 0.41]
Peer problems	3.27 (1.56)	3.29 (1.42)	2.87 (1.53)	3.66 (1.78)	0.050^*^	−0.77 [−1.55, −0.00]	3.25 (1.46)	3.58 (1.40)	2.82 (1.63)	3.82 (1.88)	0.269	−0.67 [−1.87, 0.53]	3.30 (1.69)	2.93 (1.39)	2.90 (1.48)	3.52 (1.72)	0.056	−0.99 [−2.00, 0.03]
Total difficulties score	12.24 (4.78)	11.06 (5.24)	10.97 (5.49)	12.76 (7.34)	0.040^*^	−2.97 [−5.80, −0.15]	12.22 (4.36)	11.61 (5.41)	11.53 (6.52)	12.59 (7.73)	0.396	−1.67 [−5.59, 2.25]	12.27 (5.31)	10.40 (5.03)	10.52 (4.61)	12.90 (7.20)	0.050^*^	−4.25 [−850, 0.00]
Prosocial behavior	6.30 (2.05)	6.85 (2.27)	6.58 (2.31)	6.39 (1.98)	0.187	0.73 [−0.36, 1.82]	6.31 (1.93)	6.42 (2.35)	6.76 (2.28)	6.82 (1.95)	0.946	0.05 [−1.50, 1.61]	6.30 (2.21)	7.37 (2.09)	6.43 (2.38)	6.05 (1.99)	0.071	1.45 [−0.13, 3.03]

*FW, family workshop; ES, effect size, calculated as (d value in FW group) – (d value in control group); CI = confidence interval of the ES*.

a *Data are the means (SD)*.

* *P < 0.05, FW vs. control, assessed by ANOVA for repeated measurement*.

### Effects of Intervention on SDQ Scores by Age

In order to further analyze the results in different ages, participants were stratified into two groups (3–6 and 7–9 years) (Table 5). Among the 3 to 6 year group, compared with the controls, scores of emotional symptoms subscale [−2.031 (95% CI, −3.616 to −0.447), *P* = 0.013], peer problems subscale [−1.594 (95% CI, −2.824 to −0.364), *P* = 0.012], and TDS subscale [−4.500 (95% CI, −8.598 to −0.402), *P* = 0.032] decreased significantly in the intervention group after 6 month intervention. Interestingly, subscale scores of hyperactivity–inattention, peer problem, and TDS were found statistically significant decreased in boys (all *P* < 0.05), while scores of emotional symptoms subscale decreased in girls [−2.286 (95% CI, −4.554 to −0.018), *P* = 0.048] after intervention. However, in 7 to 9 year group, there were no significant changes of SDQ subscales scores between baseline and end line.

**Table 5:** Comparison of SDQ scores between FW and control groups in participants with age groups of 3–6 and 7–9 years, respectively^a^.

	**Total**				***P***	**ES [95% CI]**	**Boys**				***P***	**ES [95% CI]**	**Girls**				***P***	**ES [95% CI]**
	**FW**		**Control**				**FW**		**Control**				**FW**		**Control**			
	**Baseline**	**6 months**	**Baseline**	**6 months**			**Baseline**	**6 months**	**Baseline**	**6 months**			**Baseline**	**6 months**	**Baseline**	**6 months**		
**3–6 years**	**(*****n=*** **32)**		**(*****n=*** **16)**				**(*****n=*** **19)**		**(*****n=*** **9)**				**(*****n=*** **13)**		**(*****n=*** **7)**			
Emotional symptoms	3.28 (1.89)	2.13 (2.03)	2.50 (1.97)	3.37 (2.58)	0.013^*^	−2.03 [−3.62, −0.45]	3.26 (1.97)	2.00 (2.21)	2.78 (2.05)	3.33 (2.739)	0.122	−1.82 [−4.16, 0.52]	3.31 (1.84)	2.31 (1.80)	2.14 (1.95)	3.43 (2.43)	0.048^*^	−2.29 [−4.55, −0.02]
Conduct problems	1.91 (1.67)	1.94 (1.87)	2.50 (1.79)	2.44 (2.07)	0.893	0.09 [−1.30, 1.49]	1.89 (1.56)	1.89 (1.82)	2.89 (1.83)	2.44 (2.007)	0.565	0.44 [−1.12, 2.01]	1.92 (1.89)	2.00 (2.00)	2.00 (1.73)	2.43 (2.30)	0.791	−0.35 [−3.11, 2.40]
Hyperactivity–inattention	4.31 (1.91)	3.28 (2.16)	4.69 (2.06)	4.62 (2.13)	0.140	−0.97 [−2.27, 0.33]	4.47 (1.65)	3.05 (1.84)	4.67 (2.35)	4.78 (2.438)	0.023^*^	−1.53 [−2.83, −0.23]	4.08 (2.29)	3.62 (2.60)	4.71 (1.80)	4.43 (1.81)	0.892	−0.18 [−2.85, 2.50]
Peer problems	3.50 (1.78)	3.28 (1.37)	2.75 (1.73)	4.12 (2.03)	0.012^*^	−1.59 [−2.82, −0.36]	3.53 (1.50)	3.47 (1.35)	2.33 (1.94)	4.33 (2.12)	0.013^*^	−2.05 [−3.63, −0.48]	3.46 (2.18)	3.00 (1.41)	3.29 (1.38)	3.86 (2.04)	0.313	−1.03 [−3.12, 1.06]
Total difficulties score	13.00 (5.29)	12.44 (6.29)	10.63 (5.48)	14.56 (7.35)	0.032^*^	−4.50 [−8.60, −0.40]	13.16 (4.63)	10.42 (5.81)	12.67 (7.37)	14.89 (7.85)	0.039^*^	−4.96 [−9.66, −0.26]	12.77 (6.31)	10.92 (5.19)	12.14 (5.15)	14.14 (7.24)	0.328	−3.83 [−11.88,4.19]
Prosocial behavior	5.84 (2.11)	7.06 (2.37)	6.06 (1.61)	6.63 (1.67)	0.397	0.66 [−0.89, 2.20]	5.84 (1.95)	6.32 (2.43)	6.56 (1.59)	6.56 (1.94)	0.644	0.47 [−1.61, 2.56]	5.85 (2.41)	8.15 (1.86)	5.43 (1.51)	6.71 (1.38)	0.353	1.02 [−1.23, 3.28]
**7–9 years**	**(*****n=*** **34)**		**(*****n=*** **22)**				**(*****n=*** **17)**		**(*****n=*** **8)**				**(*****n=*** **17)**		**(*****n=*** **14)**			
Motional symptoms	2.32 (1.61)	2.15 (1.69)	2.23 (1.88)	2.32 (2.44)	0.909	−0.27 [−1.75, 1.22]	2.47 (1.77)	2.29 (1.80)	2.00 (2.27)	1.63 (2.83)	0.888	0.20 [−2.69, 3.09]	2.18 (1.47)	2.00 (1.62)	2.33 (1.63)	2.60 (2.17)	0.520	−0.53 [−2.21, 1.14]
Conduct problems	2.18 (1.77)	2.35 (2.09)	1.41 (1.14)	2.32 (2.50)	0.127	−0.73 [−2.14, 0.67]	2.00 (1.58)	2.76 (2.22)	1.25 (0.89)	1.88 (2.17)	0.894	0.14 [−2.01, 2.29]	2.35 (1.97)	1.94 (1.92)	1.50 (1.29)	2.57 (2.71)	0.135	−1.48 [−3.46, 0.49]
Hyperactivity –inattention	3.97 (1.71)	3.68 (1.89)	3.32 (2.10)	3.50 (2.28)	0.862	−0.48 [−1.77, 0.81]	3.76 (1.75)	4.18 (1.85)	3.63 (2.67)	3.25 (2.61)	0.422	0.79 [−1.21, 2.78]	4.18 (1.70)	3.18 (1.85)	3.14 (1.79)	3.64 (2.17)	0.089	−1.50 [−3.24, 0.24]
Peer problems	3.06 (1.30)	3.29 (1.49)	2.95 (1.40)	3.32 (1.52)	0.232	−0.13 [−1.12, 0.87]	2.94 (1.39)	3.71 (1.49)	3.38 (1.06)	3.25 (1.49)	0.310	0.89 [−0.88, 2.66]	3.18 (1.24)	2.88 (1.41)	2.71 (1.54)	3.36 (1.60)	0.112	−0.94 [−2.11, 0.23]
Total difficulties score	11.53 (4.19)	11.47 (5.04)	9.91 (4.69)	11.45 (7.22)	0.459	−1.60 [−5.60, 2.39]	11.18 (3.89)	12.94 (4.75)	10.25 (5.63)	10.00 (7.19)	0.523	2.02 [−4.40, 8.43]	11.88 (4.57)	10.00 (5.03)	9.71 (4.29)	12.29 (7.36)	0.098	−4.45 [−9.78, 0.87]
Prosocial behavior	6.74 (1.91)	6.65 (2.19)	6.95 (2.68)	6.23 (2.20)	0.284	0.64 [−0.87, 2.15]	6.82 (1.81)	6.53 (2.32)	7.00 (2.98)	7.13 (2.03)	0.733	−0.42 [−2.09, 2.93]	6.65 (2.06)	6.76 (2.11)	6.93 (2.62)	5.71 (2.20)	0.185	1.33 [−0.67, 3.34]

*FW, family workshop; ES, effect size, calculated as (d value in FW group) – (d value in control group); CI, confidence interval of the ES*.

a *Data are the means (SD)*.

* *P < 0.05, FW vs. control, assessed by ANOVA for repeated measurement*.

### Effect of Intervention on SDQ Scores According to TDS

**Figure 2:**
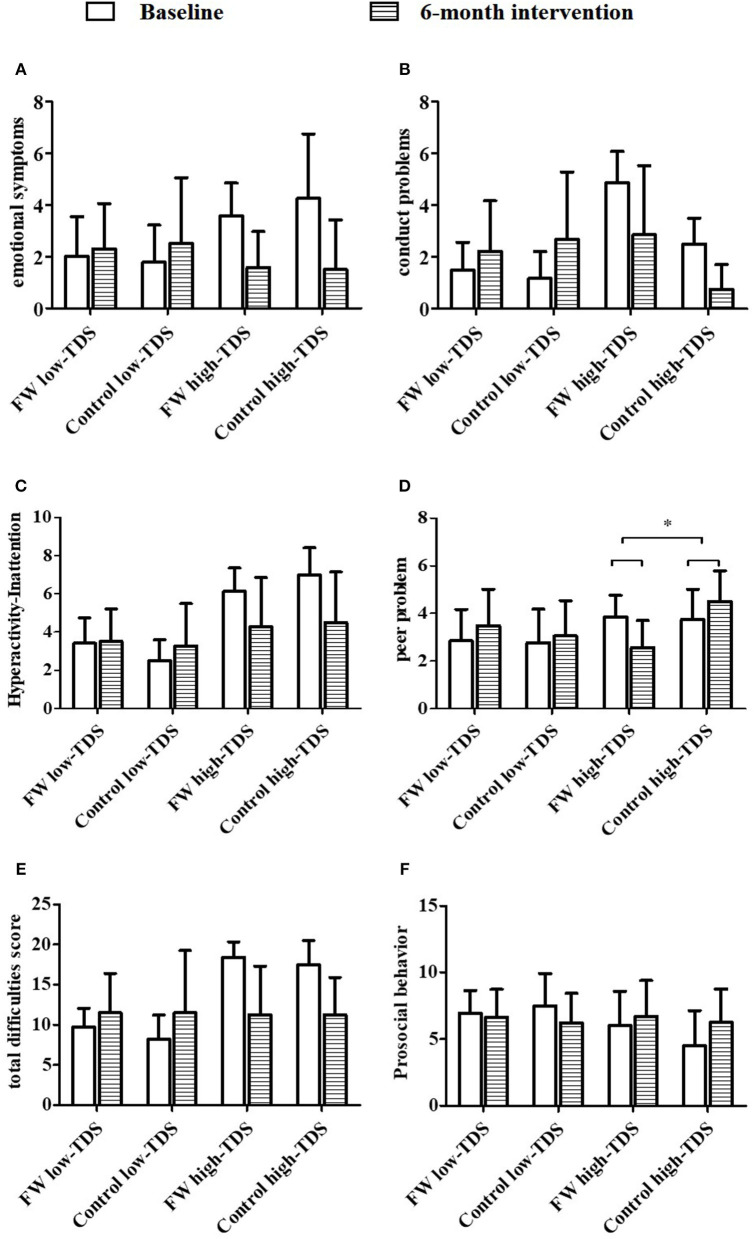
Comparison of SDQ scores between FW and control groups in participants aged 7–9 years with stratification by total difficulty score (TDS) at baseline (**A–F**, *N* = 56). FW, family workshop; Low-TDS, total difficulties score ranged from 0 to14; high-TDS, total difficulties score ranged from 15 to 40. ^*^Statistically difference between two groups with abnormal TDS (FW vs. control group), *p* < 0.05, assessed by ANOVA for repeated measurement.

In Tables 6, 7, the effect of intervention on SDQ scores was analyzed according to TDS at baseline, which was stratified as low-TDS subgroup (0–14 score) and high-TDS subgroup (15–40 score). Among total participants (Table 6), in high-TDS subgroup, statistically significant decrease was found in the score of peer problems [−2.22 (95% CI, −3.72 to 0.72), *P* = 0.005] between the intervention and control groups after intervention. No significant difference was found among participants in low-TDS subgroup between the two groups. Then, among 3 to 6 year-old participants (Table 7), in the low-TDS subgroup, score of emotional symptoms subscale decreased significantly [−1.848 (95% CI, −3.616 to −0.080), *P* = 0.041] between the two groups after intervention. At the same time, in participants aged 3–6 years in the high-TDS subgroup (*n* = 16), peer problems scores decreased significantly [−2.418 (95% CI, −4.790 to −0.047), *P* = 0.046] between the two groups after intervention. Similar trend of peer problems was found in 7- to 9-year-old participants in the high-TDS subgroup [−2.036 (95% CI, −4.051 to 0.021), *P* = 0.048, see Figure 2]. In addition, no significant changes were observed in 7 to 9 year-olds in low-TDS subgroup after the intervention.

**Table 6:** Comparison of SDQ scores between the two groups aged 3–9 years, stratified by total difficulties score at baseline^a^.

**Subscales (3–9 years old)**	**FW**		**Control**		***P***	**ES [95% CI]**
	**Baseline**	**6 months**	**Baseline**	**6 months**		
**Low TDS^b^** **(total** ***n=*** **77)**	**(*****n=*** **48)**		**(*****n=*** **29)**			
Emotional symptoms	2.17 (1.51)	2.04 (1.75)	1.62 (1.24)	2.52 (2.47)	0.082	−1.02 [−2.18, 0.13]
Conduct problems	1.27 (1.03)	1.98 (1.80)	1.38 (1.27)	2.28 (2.28)	0.713	−0.19 [−1.20, 0.83]
Hyperactivity–inattention	3.46 (1.44)	3.19 (2.04)	3.00 (1.46)	3.59 (2.16)	0.109	−0.86 [−1.91, 0.20]
Peer problems	2.92 (1.51)	3.31 (1.39)	2.45 (1.35)	3.10 (1.52)	0.560	−0.26 [−1.14, 0.62]
Total difficulties score	9.81 (2.57)	10.52 (4.88)	8.45 (2.89)	11.48 (7.12)	0.120	−2.33 [−5.28, 0.62]
Prosocial behavior	6.79 (1.80)	7.12 (2.11)	7.00 (2.24)	6.48 (1.92)	0.148	0.85 [−0.31, 2.01]
**High TDS^b^** (**total** ***n=*** **27)**	**(*****n=*** **18)**		**(*****n=*** **9)**			
Emotional symptoms	4.44 (1.46)	2.39 (2.12)	4.67 (1.80)	3.56 (2.65)	0.409	−0.94 [−3.26, 1.37]
Conduct problems	4.11 (1.45)	2.61 (2.38)	3.44 (1.24)	2.67 (2.45)	0.496	0.72 [−2.88, 1.43]
Hyperactivity–inattention	5.94 (1.39)	4.28 (1.78)	6.78 (1.39)	5.22 (2.22)	0.877	−0.11 [−1.58, 1.36]
Peer problems	4.22 (1.26)	3.22 (1.56)	4.22 (1.30)	5.44 (1.33)	0.005^*^	−2.22 [−3.72, 0.72]
Total difficulties score	18.72 (2.80)	12.50 (6.00)	19.11 (3.55)	16.89 (6.83)	0.170	−4.00 [−9.83, 1.83]
Prosocial behavior	5.00 (2.14)	6.11 (2.56)	5.22 (2.11)	6.11 (2.26)	0.867	0.22 [−2.49, 2.94]

*FW, family workshop; ES, effect size, calculated as (d value in FW group) – (d value in control group); CI, confidence interval of the ES; TDS, total difficulties score*.

a *Data are the means (SD)*.

* *P < 0.05, FW vs. control, assessed by ANOVA for repeated measurement*.

b *Low-TDS means total difficulties score ranged from 0 to 14, and high-TDS means total difficulties score ranged from 15 to 40*.

**Table 7:** Comparison of SDQ scores between FW and control groups in participants aged 3–6 years with stratification by total difficulties score at baseline^a^.

**Participants (3–6 years old)**	**FW**		**Control**		***P***	**ES [95% CI]**
	**Baseline**	**6 months**	**Baseline**	**6 months**		
**Low TDS^b^** **(total *n =* 32)**	**(*n =* 21)**		**(*n =* 11)**			
Emotional symptoms	2.38 (1.47)	1.71 (1.74)	1.36 (0.81)	2.55 (2.46)	0.041^*^	−1.85 [−3.62, −0.08]
Conduct problems	1.00 (0.90)	1.67 (1.60)	1.73 (1.56)	1.64 (1.50)	0.243	0.76 [−0.54, 2.06]
Hyperactivity–inattention	3.52 (1.60)	3.82 (1.66)	2.76 (2.39)	4.09 (2.07)	0.235	−1.04 [−2.78, 0.71]
Peer problems	3.00 (1.76)	3.10 (1.18)	1.91 (1.14)	3.18 (1.66)	0.121	−1.18 [−2.69, 0.33]
Total difficulties score	9.90 (2.91)	9.24 (4.67)	8.82 (2.79)	11.45 (6.25)	0.138	−3.30 [−7.73, 1.12]
Prosocial behavior	6.62 (1.94)	7.76 (2.00)	6.18 (1.66)	6.91 (1.30)	0.631	0.42 [−1.33, 2.16]
**High TDS^b^** (**total *n =* 16)**	**(*n =* 11)**		**(*n =* 5)**			
Emotional symptoms	5.00 (1.34)	2.91 (2.39)	5.00 (1.23)	5.20 (1.92)	0.175	−2.29 [−0.57, 1.15]
Conduct problems	3.64 (1.43)	2.45 (2.30)	4.20 (2.17)	4.20 (2.17)	0.473	−1.18 [−4.62, 2.26]
Hyperactivity–inattention	5.82 (1.54)	4.27 (1.19)	6.60 (1.52)	5.80 (1.92)	0.413	−0.75 [−2.64, 1.15]
Peer problems	4.45 (1.44)	3.64 (1.69)	4.60 (1.34)	6.20 (0.84)	0.046^*^	−2.42 [−4.79, −0.05]
Total difficulties score	18.91 (3.33)	13.27 (6.15)	20.40 (3.76)	21.40 (3.72)	0.132	−6.64 [−15.55, 2.27]
Prosocial behavior	4.36 (1.63)	5.73 (2.53)	5.80 (1.64)	6.00 (2.35)	0.489	1.16 [−2.35, 4.68]

*FW, family workshop; ES, effect size, calculated as (d value in FW group) – (d value in control group); CI, confidence interval of the ES; TDS, total difficulties score*.

a *Data are the means (SD)*.

* *P < 0.05, FW vs. control, assessed by ANOVA for repeated measurement*.

b *Low-TDS means total difficulties score ranged from 0 to 14, and high-TDS means total difficulties score ranged from 15 to 40*.

## Discussion

With the flying growth of economy, millions of children in rural China are left behind at home by their parents at a young age, resulting in various developmental disorders in both mental and behavioral development, and the lower social adaptation of LBC has become a problem of national concern (7, 38). However, interventional studies focusing on dealing with those problems in China are quite limited. Therefore, the present study investigated the effectiveness of a 6 month community-based family workshop intervention on the social adaptation of rural LBC aged 3–9 years and found that this intervention produced significant benefits for improving social adaptation of total LBC, whereas this mentioned significant effect was observed only in LBC aged 3–6 years, but not in other age groups. Moreover, the change of the subscales of social adaptation differed in two sexes: significant improvement on TDS, peer problems, and hyperactivity–inattention were found in boys, whereas emotional symptoms in girls. Furthermore, significant improvement on peer problems was observed in the intervention group with high score of TDS.

In the present study, we found that community-based family workshop intervention improved some aspects of social adaptation of LBC aged 3 to 9 years, including emotional symptoms, peer problems, and TDS (Table 4). These results partly comply with the previous studies investigating the effects of parenting training of preschooler on children's development. For instance, Cunningham et al. found great improvements in a community-based parenting program on preschoolers' behavioral problems (24), and Graziano and Hart found that parent training can effectively improve preschoolers' academic achievement, emotion regulation abilities, and executive function (39). Besides, Dunsmore and Karn found those children who can control their emotions, tended to make better decisions and exercise more judgment when interact with others (40). And the experience of parent–child interaction, peer interaction, and peer experience during the intervention can promote children's social engagement and peer competence (41).

However, our data also showed that the intervention effects on social adaptation differed by age. Remarkable improvement on emotional symptoms, peer problems, and TDS was observed among preschoolers aged 3–6 years, whereas no significant improvement was found in participants aged 7–9 years. Previous studies also showed that early parenting program had positive effects on improving emotional and behavioral adjustment in preschoolers (22, 24), which indicated that the manner of community-based family workshop intervention is much more suitable to be implemented in preschool LBC (3–6 years). Early childhood (0–6 years) is the critical period for the establishment of secure attachment, which can support the development of social adaptation, such as socially acceptable and self-control conduct in childhood and adulthood (42). As children move into school-age period, their dependence on parents declines gradually, while their social networks become broader, in which schoolmates and teachers become more and more important (43, 44). And their behaviors and emotions are more likely to be affected by school experience (45), such as heavy academic pressure, which may partly explain why no significant effect on school-aged children's social adaptation was observed after the intervention.

Moreover, we found that the effect of intervention on social adaptation was different in boys and girls. Compared with the controls, the score of TDS, peer problems, and hyperactivity–inattention decreased significantly after intervention in boys aged 3–6 years, whereas emotional symptoms improved significantly in girls. The possible mechanisms of these differences might be that boys under stress tend to have higher adrenocortical responses and greater behavioral reactivity (46). Meanwhile, compared with girls, boys have higher activity levels, lower language and self-control ability, and more displays of negative emotion (47). Additionally, parents and society tended to encourage boys to dampen their tender emotions, but to express externalizing emotions such as anger and aggressiveness, which would result in more conflicts on emotion and behavior. In contrast, girls are believed to have more communicative skills and large vocabularies and encouraged to express their emotions (47). The reasons mentioned above might explain the different improvement on behaviors and emotions between boys and girls in the present study.

TDS, as one component of SDQ, can reflect the whole condition of behaviors, emotions, and relationship. Series of studies have reported that high TDS indicates higher risk of mental disorders (35). Therefore, we evaluated the effectiveness of present intervention according to different levels of TDS (low TDS and high TDS). Interestingly, significant improvement on peer problems was observed in the intervention group with high TDS. Many studies have reported that healthy peer relationship originates from the childhood experiences of being loved, valued, and supported by his parents (48). Thus, family workshop intervention containing considerable activities and education on parent–child interaction in the present study may help children with high TDS improve their peer relationship significantly.

There are some limitations in the present study. First, we used a comprehensive intervention that combined integrated intervention and parent–child activity, rather than an independent design in which the combination could be directly compared with a parenting program and a parent–child interaction program, because such a design could find out the key ingredients of the intervention. However, given the interaction between parenting knowledge and parent–child interaction, we found little justification for not using the comprehensive pattern. Second, only parent-reported questionnaire of children was used as outcome measures in the present study. It is still unclear with the change of the caregivers or the relationship between caregiver/parents and child. Other forms of measurement such as observation of children's behavior, the tests of parent–child interaction, and tests of caregiver's knowledge of parenting knowledge could be used in future study. Third, we did not follow up the long-term effect in LBC after completing the 6 month intervention. It is still unclear how long the benefits of the intervention will last in later life of those LBC. Thus, further studies are needed to assess the long-term effect of the community-based family workshop.

## Conclusions

In the present study, we found that community-based family workshop intervention can improve social adaptation among total rural LBC, especially in those aged 3–6 years. Furthermore, the effect of this intervention was different in boys and girls and also influenced by age and TDS. Results of the present study indicate that community-based family workshop intervention is a useful way to improve mental and behavioral health in rural LBC, which could be applied in other rural community-based setting in China in the future.

## Data Availability Statement

The datasets generated for this study are available on request to the corresponding author.

## Ethics Statement

The studies involving human participants were reviewed and approved by the institutional research ethics committee of Sun Yat-sen University and the institutional review board of Guangdong Women's Federation. Written informed consent to participate in this study was provided by the participants' legal guardian/next of kin.

## Author Contributions

LG, ML, and YZ contributed conception and design of the study. XZ and ML organized the database. XZ performed the statistical analysis. XZ wrote the first draft of the manuscript. ML, LG, and YZ wrote sections of the manuscript. All authors contributed to manuscript revision, read, and approved the submitted version.

## Conflict of Interest

The authors declare that the research was conducted in the absence of any commercial or financial relationships that could be construed as a potential conflict of interest.

## Abbreviations

LBC: left-behind children
SDQ: the Strength and Difficulties Questionnaire
TDS: total difficulties score
FW: family workshop.
